# Changes in serum TG levels during pregnancy and their association with postpartum hypertriglyceridemia: a population-based prospective cohort study

**DOI:** 10.1186/s12944-021-01549-y

**Published:** 2021-09-29

**Authors:** Yandi Zhu, Haiyan Zhu, Qinyu Dang, Qian Yang, Dongxu Huang, Yadi Zhang, Xiaxia Cai, Huanling Yu

**Affiliations:** 1 Department of Nutrition and Food Hygiene, School of Public Health, Capital Medical University, 100069 Beijing, P.R. China; 2 FuXing Hospital, Capital Medical University, 100045 Beijing, P.R. China

**Keywords:** TG, Pregnancy, Postpartum, Hypertriglyceridemia, Cut-off, Pre-BMI, GDM

## Abstract

**Background:**

Blood lipid increases during gestation are considered a physiological adaption, and decrease after delivery. However, some adverse pregnancy outcomes are thought to be related to gestational lipid levels. Therefore, it is necessary to have a reference range for lipid changes during gestation. The present study aims to describe triglyceride (TG) changes during pregnancy and 42 days postpartum and to find cut-off points for TG levels during the first, second, and third trimesters.

**Methods:**

A total of 908 pregnant women were followed from recruitment to 42 days postpartum, and their serum lipids were collected at gestational weeks 6–8, 16, 24, and 36 and 42 days postpartum. The major outcome was postpartum hypertriglyceridemia. The association between gestational and postpartum TG levels was analysed by stepwise multiple linear regression. A two-stage approach including a linear mixed-effect model and linear or logistic regression was conducted to explore the contribution of the changes in TG over time in pregnancy to postpartum hypertriglyceridemia. Logistic regression was constructed to examine the association between gestational TG levels and postpartum hypertriglyceridemia. Cut-off points were calculated by receiver operating characteristic (ROC) curves.

**Results:**

There was a tendency for serum TG to increase with gestational age and decrease at 42 days postpartum. Prepregnancy overweight, obesity, and GDM intensified this elevation. Higher TG levels at gestational weeks 6–8, 16, 24, and 36 were positively associated with a higher risk of postpartum hypertriglyceridemia [OR 4.962, 95 % CI (3.007–8.189); OR 2.076, 95 % CI (1.303–3.309); OR 1.563, 95 % CI (1.092–2.236); and OR 1.534, 95 % CI (1.208–1.946), respectively]. The trend of the change in TG over time was positively associated with the TG level and risk of postpartum hypertriglyceridemia [OR 11.660, 95 % CI (6.018–22.591)]. Based on ROC curves, the cut-off points of serum TG levels were 1.93, 2.35, and 3.08 mmol/L at gestational weeks 16, 24, and 36, respectively. Stratified analysis of prepregnancy body mass index (pre-BMI) and GDM showed that higher gestational TG was a risk factor for postpartum hypertriglyceridemia in women with normal pre-BMI and without GDM.

**Conclusions:**

Gestational TG and its elevation were risk and predictive factors of postpartum hypertriglyceridemia, especially in pregnant women with normal pre-BMI or without GDM.

**Supplementary Information:**

The online version contains supplementary material available at 10.1186/s12944-021-01549-y.

## Background

Maternal lipid increases throughout pregnancy are thought to be a physiological response to gestation [1]. During gestation, maternal triglyceride (TG) levels gradually increase more than three times from the week 12 gestation to the third trimester [1, 2] to support maternal energy storage and fetal growth [3]. However, higher serum TG levels in pregnancy are closely associated with the risk of pregnancy complications such as gestational diabetes mellitus (GDM) [4], preeclampsia (PE) [5], and intrahepatic cholestasis of pregnancy (ICP) [6] and adverse pregnancy outcomes such as preterm birth [2], large for gestational age (LGA) infants [7], and macrosomia [8].

After delivery, maternal organs and systems usually require approximately 42 days to return to or approach a normal state of nonpregnancy, also known as puerperium. Nevertheless, studies found that elevated TGs in pregnancy were related to postpartum abnormal glucose metabolism [9]. Adank et al. found that high maternal TG in gestation was positively associated with blood pressure and sustained hypertension even 6 and 9 years after pregnancy [10]. Moreover, a high TG concentration has been proven to be an independent risk factor for cardiovascular diseases (CVDs), such as coronary artery disease and cardiomyovasculopathy [2, 11, 12]. Pregnancy may result in long-term lipid profile alteration or reset in women [13]. Disorders of lipid metabolism can be long-lasting, and the potential risk of CVD and chronic metabolic diseases may threaten women’s long-term health if elevated TGs in pregnancy cannot return to normal after delivery.

However, there is no consensus reference range for maternal TG levels during gestation. Wang et al. [14] took adverse pregnancy outcomes as the outcome and selected the 95th percentiles to recommend that the TG reference value should be less than 1.95 mmol/L and 3.56 mmol/L in early and middle pregnancy, respectively. Given the lack of trimester-specific reference values and postpartum cut-off points, we designed this study to characterize changes in serum TG levels throughout pregnancy and after delivery and to investigate the association between gestational TGs and the risk of persistent postpartum hypertriglyceridemia. The serum TG cut-off points in the first, second, and third trimesters of gestation were calculated based on the risk of postpartum hypertriglyceridemia.

## Methods

### Study design and population

**Fig. 1:**
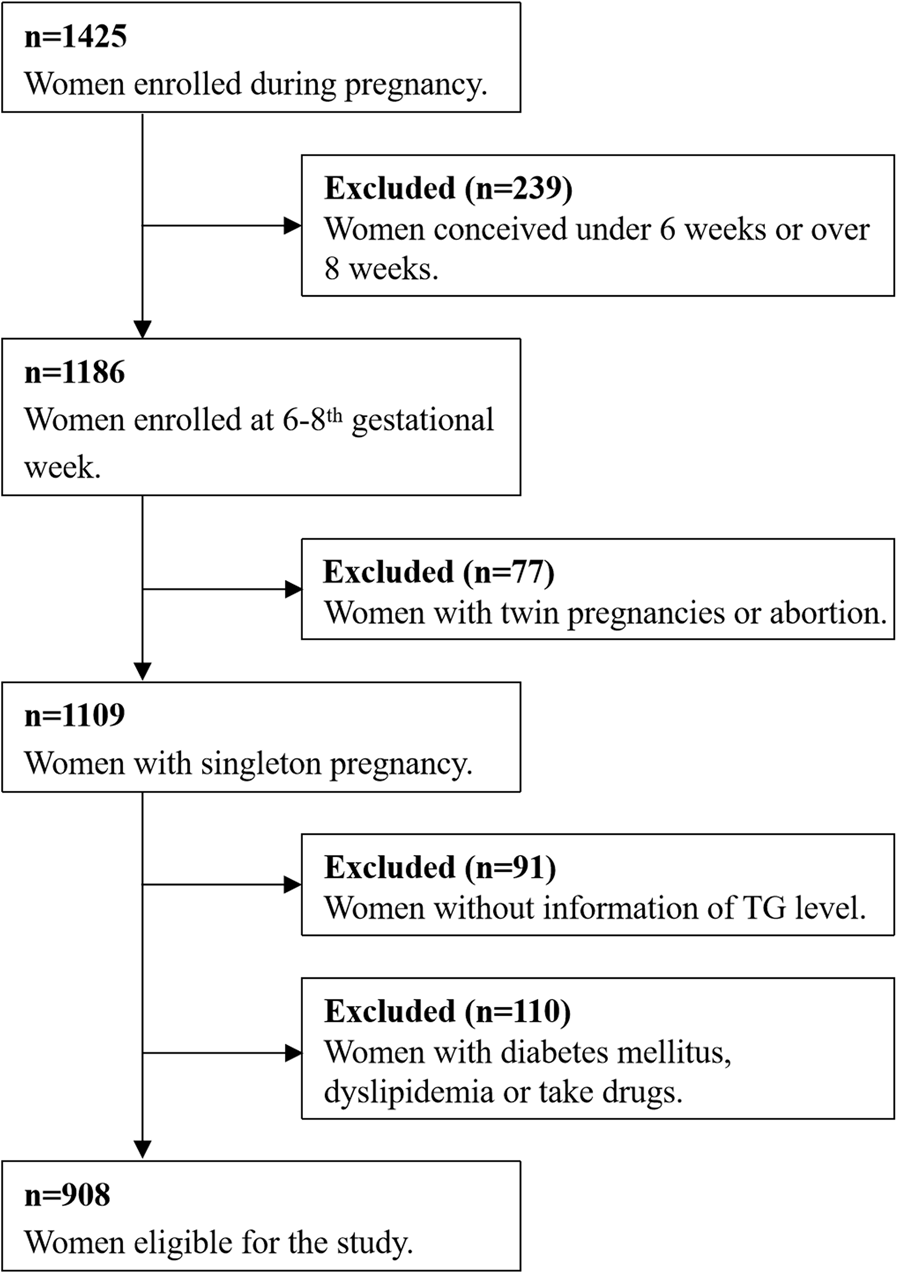
Flow chart of the study population

The prospective cohort study was conducted in FuXing Hospital, Capital Medical University in Beijing, China. Participants who received regular prenatal care and delivered from November 2018 to January 2020 were recruited. The inclusion criteria were as follows: (1) naturally conceived; (2) singleton pregnancy; and (3) gestational age 6–8 weeks at enrolment. The exclusion criteria were as follows: (1) women with diabetes mellitus before gestation; (2) women with autoimmune disease; and (3) women who took drugs affecting lipid metabolism. Ultimately, 908 pregnant women were included in the study (Fig. 1) and were evaluated at five follow-up periods: gestational weeks 6–8 (baseline), 16, 24 and 36, and 42 days postpartum. This study was approved by the Ethics Committee of Capital Medical University (2012SY29) and abided by the Declaration of Helsinki principles. All participants provided written informed consent at study enrolment.

### Data collection

The demographic characteristics and baseline information included maternal age, prepregnancy height and weight, weight at delivery, parity, blood pressure, disease history (cardiovascular disease, diabetes mellitus, chronic hypertension, etc.), smoking and drinking habits, degree of education, and profession, and this information was obtained at recruitment. Pre-BMI was regarded as weight (kilograms) per square height (meter). According to Chinese cut-off points [15], participants were divided into four groups: low weight (pre-BMI < 18.5 kg/m^2^), normal weight (18.5 kg/m^2^ ≤ pre-BMI ≤ 23.9 kg/m^2^), overweight (24.0 kg/m^2^ ≤ pre-BMI ≤ 27.9 kg/m^2^), or obese (pre-BMI ≥ 28.0 kg/m^2^). Gestational weight gain (GWG) was calculated using prepregnancy weight and weight at delivery. Gestational age was defined by the combination of the last menstrual period and ultrasound in the early first trimester. According to the criteria of the International Association of the Diabetes and Pregnancy Study Groups (IADPSG) [16], GDM was diagnosed when one or more of the following indices were met the 75 g Oral Glucose Tolerance Test (OGTT): fasting glucose level ≥ 5.10 mmol/L, 1-h glucose level ≥ 10.00 mmol/L, and 2-h glucose level ≥ 8.50 mmol/L.

### Lipid measurement

Fasting blood samples were measured at gestational weeks 6–8, 16, 24, and 36, and random blood samples were measured at 42 days postpartum. Serum TG, total cholesterol (TC), low-density lipoprotein cholesterol (LDL-C), high-density lipoprotein cholesterol (HDL-C), and glucose levels were measured by a Hitachi type 7180 automatic biochemical analyser (Yokohama, Japan) at the clinical laboratory of Fuxing Hospital affiliated with Capital Medical University.

### Outcomes

The major outcome was hypertriglyceridemia at 42 days postpartum. As the diagnostic criteria of dyslipidemia in Chinese adults are not suitable for random blood samples of pregnant women, hypertriglyceridemia was defined as a serum TG level ≥ the 75th percentile (P75) of all participants at 42 days postpartum.

### Statistical analysis

Stata 16.1 (Stata Corp, TX, USA) and SPSS software 25.0 (SPSS, Chicago, IL, USA) were used to conduct all statistical analyses. Continuous variables are presented as the mean ± SD, while categorical variables are presented as numbers (proportion). The comparisons of TG levels among different periods or groups were performed by one-way ANOVA. The Bonferroni test was used for pairwise comparisons if homogeneity of variance was satisfied, and the Tamhane T2 test was used otherwise.

Pearson correlation and stepwise multiple linear regression models were used to explore the associations between TG levels at sampling weeks and 42 days postpartum. The TG level at 42 days postpartum was the dependent variable. The TG level at each gestational week, baseline TG, age, GWG, pre-BMI, GDM, and gestational age at delivery were independent variables. Because the TG level generally did not increase before the gestational week 12, the TG level at gestational week 6–8 was regarded as the baseline TG level. The variable was entered into the model if α ≤ 0.05 and was ruled out if α ≥ 0.10. Considering the longitudinal design, a two-stage approach [17] was conducted to explore the association between the trend of the change in TG over time throughout pregnancy and the TG level or postpartum hypertriglyceridemia. First, a linear mixed-effect model (LME) was carried out for TG level including gestational week at sampling as fixed and random effects and predicted the best linear unbiased predictor (BLUP) of random intercepts and slopes. The predicted intercept referred to the mean TG level at gestational weeks 6–8, and the predicted slope referred to the trend of the TG level change over time during gestation. In the second stage, the BLUP estimates of the TG intercepts and slopes were used as continuous predictors in linear and logistic regression models, respectively, taking TG level at 42 days postpartum and hypertriglyceridemia as the outcomes. A logistic regression model was constructed to explore the association between gestational TG level and postpartum hypertriglyceridemia and to evaluate the odds ratios (ORs) and 95 % confidence intervals (95 % CIs). The model was adjusted for baseline TG, age, GWG, pre-BMI, and GDM.

Cut-off points of serum TG level at studied gestational weeks were selected by the point nearest to the top-left most corner of the receiver operating characteristic (ROC) curves, which was assessed by the maximum value of Youden Index [18]. Sensitivity, specificity, and 95 % CIs were calculated. The area under the ROC curve (AUC) was used to evaluate the discrimination of the predictive model. The Hosmer–Lemeshow goodness-of-fit test was carried out for the assessment of calibration [19]. A hierarchical logistic regression model was used for subgroup analysis among different pre-BMI subgroups and between GDM subgroups. A *P* value of < 0.05 was considered statistically significant.

## Results

### Characteristics of study population

Table 1 shows the characteristics of the study population. Only two participants had a history of alcohol consumption, and none were smokers.

**Table 1:** Characteristics of study population

Characteristics	Mean ± SD or N (%)
Age, years	31.8 ± 3.9
Height, cm	162.94 ± 4.98
Weight, kg	
Prepregnancy weight, kg	59.18 ± 10.01
Weight at delivery, kg	71.43 ± 10.17
Gestational weight gain, kg	12.57 ± 4.92
Prepregnancy BMI, kg/m^2^	22.37 ± 3.37
< 18.5	47 (5.2)
18.5–23.9	361 (39.8)
24.0–27.9	125 (13.8)
≥ 28.0	31 (3.4)
Missed	344 (37.8)
GDM	233 (25.7)
Parity	
Primiparas	617 (68.0)
Multiparas	291 (32.0)
Gestational age at delivery, week	39.3 ± 1.4

Data are presented as mean ± SD or n (%)

*BMI* body mass index, *GDM* gestational diabetes mellitus

### Longitudinal change in serum TG levels of pregnant women during gestation and after delivery

**Fig. 2:**
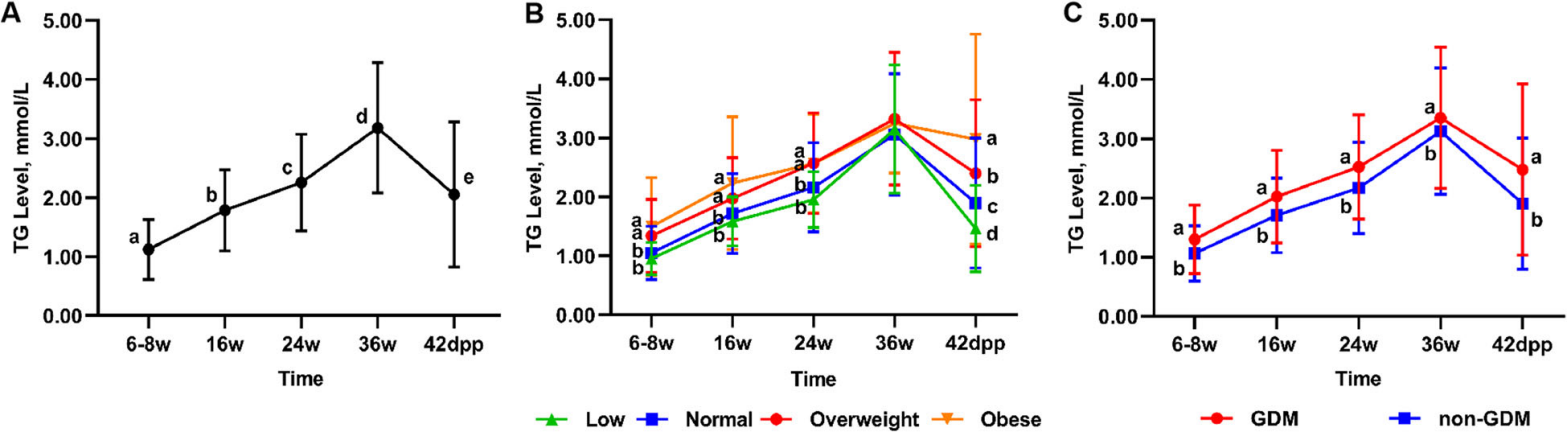
Longitudinal change in serum TG levels of pregnant women during gestation and after delivery.** A-** Total participants, **B-** Comparison among pregnant women with different pre-BMIs, **C-** Comparison between pregnant women with or without GDM. The x-axis represents different periods, including gestational weeks 6–8, 16, 24, and 36 and 42 days postpartum. The y-axis indicates serum TG level of pregnant women. The dots and error bars represent the mean ± SD of TG levels. Different letters represent the significance of significant differences in each period or between each subgroup. A *P* value < 0.05 indicates statistical significance

There was a tendency for serum TG to increase with gestational age. In particular, TG levels increased 3-fold from the first to third trimesters and decreased at 42 days postpartum (Fig. 2 A). The cut-off point of postpartum hypertriglyceridemia defined by the P75 of random TG level at 42 days postpartum was 2.66 mmol/L.

Compared with the low weight and normal weight groups, the TG levels of the overweight and obese groups were significantly higher at gestational weeks 6–8, 16, and 24 (Fig. 2B). At 42 days postpartum, significant differences existed among all four groups, while the obese group had the highest mean serum TG. The TG levels of pregnant women with GDM were consistently higher than those of the non-GDM group throughout gestation and at 42 days postpartum (Fig. 2 C).

### Associations between serum TG levels during pregnancy and at 42 days postpartum

The TG level at 42 days postpartum was significantly positively correlated with the TG level at gestational weeks 6–8, 16, 24, and 36 according to Pearson correlation analysis (Table S1 in the Supplementary Appendix). After adjustment for age, GWG, pre-BMI, GDM, and gestational age at delivery, the TG level at 42 days postpartum was found to be positively associated with the TG level at gestational week 6–8 (baseline) (B = 0.789, *P* < 0.001). After adjustment for baseline TG, age, GWG, pre-BMI, GDM, and gestational age at delivery, positive associations between TG level at 42 days postpartum and respective TG level at gestational weeks 16, 24, and 36 were found by stepwise multiple linear regression (B = 0.430, *P* < 0.001; B = 0.369, *P* < 0.001; B = 0.244, *P* < 0.001) (Table 2).

**Table 2:** Stepwise multiple linear regression for gestational TG level and 42 days postpartum

Time	Variables	B (SE)	95 % CI	β	*P*
Week 6–8 (Baseline)	Constant	-0.858 (0.360)	-1.565–-0.151		0.017
	TG	0.789 (0.094)	0.605–0.973	0.355	**< 0.001**
	Pre-BMI	0.069 (0.015)	0.040–0.098	0.200	< 0.001
	GDM	0.440 (0.113)	0.218–0.662	0.160	< 0.001
	GWG	0.025 (0.010)	0.006–0.044	0.104	0.011
Week 16	Constant	-0.960 (0.362)	-1.672–-0.249		0.008
	TG	0.430 (0.092)	0.249–0.612	0.263	**< 0.001**
	Pre-BMI	0.053 (0.015)	0.023–0.082	0.148	< 0.001
	Baseline TG	0.424 (0.123)	0.182–0.666	0.196	0.001
	GWG	0.033 (0.010)	0.014–0.052	0.139	0.001
	GDM	0.376 (0.110)	0.159–0.593	0.140	0.001
Week 24	Constant	-1.135 (0.358)	-1.838–-0.433		0.002
	TG	0.369 (0.077)	0.217–0.520	0.242	**< 0.001**
	Pre-BMI	0.060 (0.014)	0.032–0.089	0.174	< 0.001
	Baseline TG	0.472 (0.117)	0.242–0.701	0.207	< 0.001
	GDM	0.414 (0.111)	0.195–0.633	0.151	< 0.001
	GWG	0.026 (0.010)	0.007–0.045	0.109	0.007
Week 36	Constant	-1.398 (0.366)	-2.117–-0.678		< 0.001
	TG	0.244 (0.050)	0.146–0.341	0.214	**< 0.001**
	Baseline TG	0.573 (0.105)	0.366–0.780	0.253	< 0.001
	Pre-BMI	0.071 (0.014)	0.043–0.099	0.205	< 0.001
	GDM	0.452 (0.110)	0.235–0.669	0.165	< 0.001
	GWG	0.022 (0.010)	0.004–0.041	0.094	0.022

Dependent variable: serum TG level at 42 days postpartum

Independent variables: serum TG level at each gestational week, baseline TG (TG of week 6–8), age, GWG, pre-BMI, GDM, and gestational age at delivery

If α ≤ 0.05, the variable was entered into the model, and the variable was ruled out if α ≥ 0.10

*B* unstandardized linear regression coefficient, *β* standardized linear regression coefficient

*SE* standard error, *TG* triglyceride, *GWG* gestational weight gain, *Pre-BMI* prepregnancy BMI, *GDM* gestational diabetes mellitus

Serum TG levels during pregnancy were longitudinally time-varying. According to the LME and linear regression model, the trend of the change in TG levels over time, which was evaluated by the TG rate of change per gestational week (slope), showed a positive association with TG levels at 42 days postpartum [B 1.425, 95 % CI (1.162–1.688)] (Table 3).

**Table 3:** The association between the gestational TG change trend and serum TG levels at 42 days postpartum

	B (SE)	95 % CI	*P*
TG			
Slope	1.425 (0.134)	1.162–1.688	**< 0.001**
Intercept	0.512 (0.052)	0.410–0.614	**< 0.001**
Age	0.014 (0.006)	0.002–0.027	0.021
GWG	0.024 (0.005)	0.014–0.033	< 0.001
Pre-BMI	0.067 (0.007)	0.053–0.081	< 0.001
GDM	0.443 (0.055)	0.335–0.550	< 0.001
Gestational age at delivery	-0.011 (0.022)	-0.054–0.031	0.602

The model was adjusted for age, GWG, pre-BMI, GDM and gestational age at delivery

*B* longitudinal linear regression coefficient

*SE* standard error, *TG* triglyceride, *GWG* gestational weight gain, *Pre-BMI* prepregnancy BMI, *GDM* gestational diabetes mellitus

### Associations between serum TG level during pregnancy and risk of postpartum hypertriglyceridemia

After adjustment for age, GWG, pre-BMI, and GDM, logistic regression analysis showed that a higher TG level at gestational weeks 6–8 (baseline) was positively associated with a higher risk of hypertriglyceridemia at 42 days postpartum [OR 4.962, 95 % CI (3.007–8.189)]. After adjustment for baseline TGs, age, GWG, pre-BMI, and GDM, higher TG levels at gestational weeks 16, 24, and 36 were found to be positively associated with a higher risk of postpartum hypertriglyceridemia [OR 2.076, 95 % CI (1.303–3.309); OR 1.563, 95 % CI (1.092–2.236); OR 1.534, 95 % CI (1.208–1.946), respectively] **(**Table 4**)**.

**Table 4:** Logistic regression for the risk of postpartum hypertriglyceridemia

		Week 6–8			Week 16			Week 24			Week 36		
		OR	95 % CI	*P*	OR	95 % CI	*P*	OR	95 % CI	*P*	OR	95 % CI	*P*
TG		**4.962**	3.007–8.189	**< 0.001**	**2.076**	1.303–3.309	**0.002**	**1.563**	1.092–2.236	**0.015**	**1.534**	1.208–1.946	**< 0.001**
Baseline TG					2.696	1.441–5.044	0.002	3.088	1.691–5.637	< 0.001	3.132	1.805–5.436	< 0.001
Age		1.045	0.982–1.111	0.166	1.041	0.975–1.112	0.224	1.053	0.989–1.120	0.106	1.063	0.997–1.133	0.060
GWG		1.056	1.008–1.106	0.023	1.073	1.022–1.127	0.005	1.060	1.011–1.110	0.015	1.053	1.004–1.105	0.033
pre-BMI		1.136	1.059–1.218	< 0.001	1.112	1.032–1.198	0.005	1.128	1.051–1.209	0.001	1.149	1.070–1.235	< 0.001
GDM	No	Reference			Reference			Reference			Reference		
	Yes	1.712	1.022–2.868	0.041	1.787	1.047–3.048	0.033	1.775	1.054–2.988	0.031	1.858	1.098–3.143	0.021

The model was adjusted for baseline TG (TG of week 6–8), age, GWG, pre-BMI, and GDM

*TG* triglyceride, *GWG* gestational weight gain, *Pre-BMI* prepregnancy BMI, *GDM* gestational diabetes mellitus

LME and logistic regression models showed that a higher TG rate of change per gestational week in pregnancy, which referred to the trend of the change in TG over time, was positively associated with a higher risk of postpartum hypertriglyceridemia [OR 11.660, 95 % CI (6.018–22.591)] **(**Table 5**)**.

**Table 5:** The association between the gestational trend of the change in TG and the risk of postpartum hypertriglyceridemia

	OR	95 % CI	*P*
TG			
Slope	11.660	6.018–22.591	**< 0.001**
Intercept	2.731	2.074–3.598	**< 0.001**
Age	1.040	1.007–1.075	0.019
GWG	1.052	1.027–1.078	< 0.001
Pre-BMI	1.140	1.099–1.181	< 0.001
GDM	1.834	1.406–2.393	< 0.001
Gestational age at delivery	0.915	0.820–1.020	0.110

The model was adjusted for age, GWG, pre-BMI, GDM, and gestational age at delivery

*TG* triglyceride, *GWG* gestational weight gain, *Pre-BMI* prepregnancy BMI, *GDM* gestational diabetes mellitus

### Cut-off points of serum TG levels during the first, second, and third trimesters

**Fig. 3:**
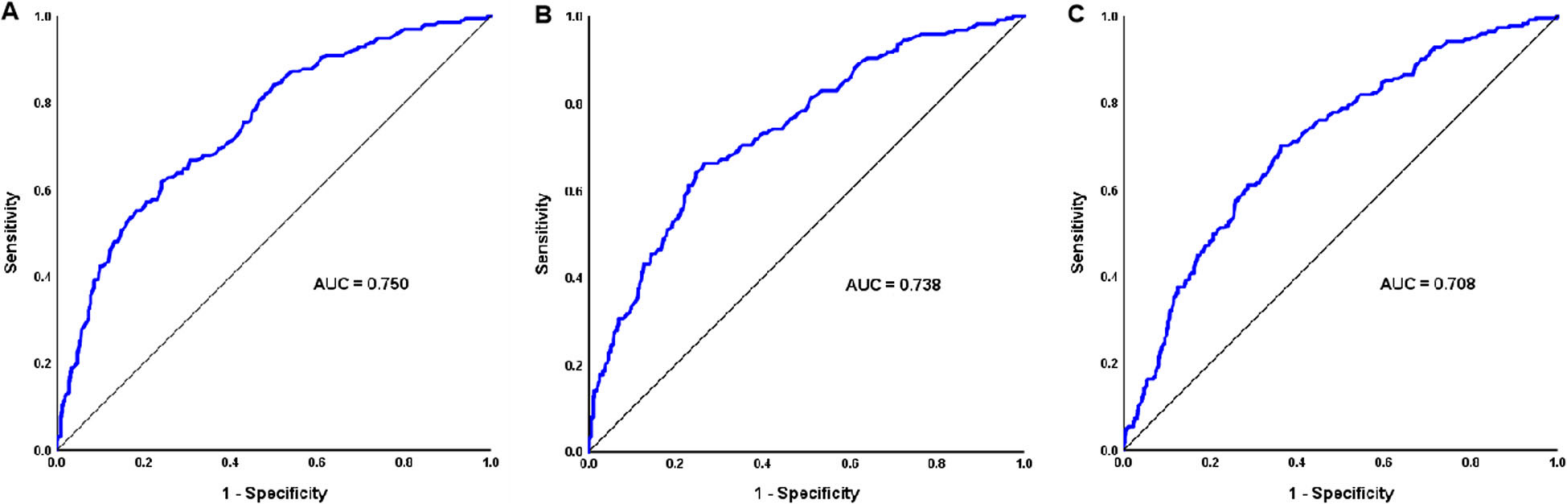
ROC curves for the prediction model of gestational TG levels in postpartum hypertriglyceridemia. **A-** Gestational week 16, **B-** Gestational week 24, **C-** Gestational week 36. The test variables of ROC curves are serum TG levels at each gestational week. The status variable of the ROC curves was hypertriglyceridemia at 42 days postpartum

According to ROC curves of the prediction model for gestational TG level on postpartum hypertriglyceridemia, optimal TG cut-off points were 1.93 mmol/L, 2.35 mmol/L, and 3.08 mmol/L at gestational weeks 16, 24, and 36, respectively, which were recommended as the reference upper limits for the first, second, and third trimesters **(**Table 6**)**. The model had strong predictive power, as the AUCs were 0.750 (95 % CI 0.711–0.789), 0.738 (95 % CI 0.699–0.776), and 0.708 (95 % CI 0.669–0.747) for gestational weeks 16, 24, and 36, respectively (Fig. 3). The Hosmer–Lemeshow goodness-of-fit test revealed favourable calibration and discrimination of the predictive model **(**Table 6**)**.

**Table 6:** Prediction model of gestational TG level on postpartum hypertriglyceridemia

	TG cut-off, mmol/L	AUC (95 % CI)	Sensitivity	Specificity	χ^2^	*P*
First trimester	1.93	0.750 (0.711–0.789)	61.7 %	75.9 %	10.382	0.239
Second trimester	2.35	0.738 (0.699–0.776)	66.2 %	73.4 %	9.786	0.280
Third trimester	3.08	0.708 (0.669–0.747)	70.1 %	63.8 %	21.917	0.050

*P* value of > 0.05 was considered as the prediction model with good calibration.

*TG* triglyceride, *AUC* area under the ROC curve

### Stratified analysis of pre-BMI and GDM on the risk of postpartum hypertriglyceridemia

According to the hierarchical logistic regression analysis for different pre-BMI subgroups, the positive association between gestational TGs and postpartum hypertriglyceridemia was no longer significant in the low weight or obese group **(**Table 7**)**. For the subgroup divided by GDM, TG levels at gestational weeks 6–8, 16, 24, and 36 were still risk factors for postpartum hypertriglyceridemia for women without GDM [OR 5.428, 95 % CI (2.800–10.523); OR 2.020, 95 % CI (1.150–3.547); OR 1.614, 95 % CI (1.070–2.434); and OR 1.702, 95 % CI (1.242–2.331), respectively] but not for women with GDM **(**Table 7**)**.

**Table 7:** Hierarchical logistic regression for the risk of postpartum hypertriglyceridemia

	Pre-BMI subgroup												GDM subgroup					
	Low weight			Normal weight			Overweight			Obese			GDM			Non-GDM		
	OR	95 % CI	*P*	OR	95 % CI	*P*	OR	95 % CI	*P*	OR	95 % CI	*P*	OR	95 % CI	*P*	OR	95 % CI	*P*
Week 6–8	0.553	0.002–140.633	0.834	**4.704**	2.472–8.950	**< 0.001**	**3.526**	1.432–8.685	**0.006**	3.913	0.999–107.717	0.051	**3.143**	1.418–6.968	**0.005**	**5.428**	2.800–10.523	**< 0.001**
Week 16	0.888	0.015–53.484	0.955	**2.530**	1.308–4.893	**0.006**	1.621	0.717–3.662	0.245	2.738	0.084–88.911	0.571	1.740	0.680–4.448	0.248	**2.020**	1.150–3.547	**0.014**
Week 24	0.668	0.016–27.395	0.832	1.551	0.929–2.590	0.094	1.749	0.942–3.248	0.077	2.519	0.185–34.310	0.488	1.394	0.623–3.120	0.420	**1.614**	1.070–2.434	**0.022**
Week 36	1.721	0.549–5.392	0.351	**1.780**	1.252–2.531	**0.001**	**1.648**	1.067–2.545	**0.024**	0.492	0.088–2.744	0.419	1.466	0.961–2.237	0.076	**1.702**	1.242–2.331	**0.001**

The model of pre-BMI subgroup was adjusted for baseline TG (TG of week 6–8), age, GWG, GDM, and gestational age at delivery

The model of GDM subgroup was adjusted for baseline TG (TG of week 6–8), age, GWG, pre-BMI, and gestational age at delivery

*Pre-BMI* prepregnancy BMI, *GDM* gestational diabetes mellitus, *GWG* gestational weight gain

## Discussion

This prospective study described the TG level change in pregnancy and after delivery. There was a tendency for maternal serum TG to increase gradually with gestational age and decrease at 42 days postpartum. In group comparisons, prepregnancy overweight and obese women and women with GDM had higher serum TG during pregnancy and after delivery than women with normal lipid levels. Serum TG during pregnancy was positively associated with TG levels at 42 days postpartum. After adjustment for baseline TG and other factors, a higher TG level during pregnancy was found to increase the risk of postpartum hypertriglyceridemia. In addition, the trend of the change in TG over time during pregnancy was also positively associated with postpartum TG levels and the risk of postpartum hypertriglyceridemia. These main results indicated that a high level of TG and its elevation during pregnancy seriously threaten postpartum lipid metabolism, especially in nonobese pregnant women or pregnant women without GDM.

During pregnancy, the increased secretion of hormones, including progesterone, prolactin, oestrogen, insulin, and human placental lactogen, in maternal blood promotes the intestinal absorption of lipids [1]. As a result, maternal blood lipids increase during pregnancy, which helps the fetus obtain lipid nutrients through the placenta to support growth and development. Several population-based studies have found that serum concentrations of TG, TC, LDL-C, and HDL-C increased to distinct degrees starting at gestational week 12 and developed more prominent increases in mid- and late pregnancy [1, 2]. The present study demonstrated that maternal serum TG increased with gestational age throughout pregnancy, which was consistent with previous findings [1, 20]. Many factors, such as pre-BMI, GWG, maternal nutrition, and TG concentration before pregnancy, were reported to affect gestational serum TG levels [21]. The present study showed that prepregnancy overweight and obese women had higher TG levels during pregnancy than low and normal pre-BMI women. Additionally, a higher TG level was found in women with GDM throughout pregnancy, which agreed with the conclusion of Herrera et al. [22].

The present study revealed that maternal serum TG levels decreased at 42 days postpartum and were equivalent to the mean level in the second trimester but were still higher than those in the first trimester. Hansen et al. [23] reported that maternal serum total lipids at three days postpartum were higher than those in the second trimester and declined at six weeks postpartum. Mbadugha et al. [24] found that maternal serum lipids dropped significantly within a week after delivery but returned to normal levels after approximately one year. Studies have indicated that the altered lipid profile following pregnancy adjustments was not easily corrected. Moreover, the present study found that the TG level of women with higher pre-BMI decreased more slowly after delivery. For prepregnancy obese women, the TG level at 42 days postpartum was still similar to that in the third trimester. Overweight or obesity originates from nutrient excess, which is always accompanied by increased serum lipids [25]. Adipose tissue expansion and insulin resistance promote the esterification of free fatty acids (FFAs) into TGs, which in turn increases fat storage and aggravates obesity [26]. The vicious cycle restrains the recovery of elevated gestational TG after delivery for obese women.

The present study indicated that TG levels during gestation and their change rate per gestational week were both risk factors for postpartum hypertriglyceridemia, especially increased levels during the first trimester. Therefore, elevated gestational TG can be a biomarker for postpartum hypertriglyceridemia. Prompted by the lack of existing research data, this study expressly set up a prediction model for postpartum hypertriglyceridemia using ROC curves and found serum TG cut-off points in pregnancy. The results recommended that maternal serum TG levels should be less than 1.93 mmol/L, 2.35 mmol/L, and 3.08 mmol/L in the first, second, and third trimesters, respectively. According to the Chinese Guideline for the Prevention and Management of Dyslipidaemia in Adults [15], the appropriate TG level for adults is recommended to be less than 1.70 mmol/L. However, this study also found that the cut-off level of TG before pregnancy should be lower than 1.12 mmol/L to prevent postpartum hypertriglyceridemia. To date, few studies have provided reference values to evaluate maternal lipid status during pregnancy. Wang et al. [14] used adverse pregnancy outcomes as the main outcome and found that TG levels should be below 1.95 mmol/L and 3.56 mmol/L in early and middle pregnancy, respectively, selected by 95th percentiles. The 95 % CI has been acknowledged to establish medical reference ranges, but for pregnant women, as a special group, the risk of postpartum outcomes should be considered to set up specific cut-offs. Therefore, although there was no exact gestational week, the 95th reference value in their study was slightly higher than that recommended in the present study, which may be due to the distinct outcomes and methods.

Based on the findings of the present study, serum TG levels should be monitored regularly during pregnancy, particularly in women with high levels of serum lipids before pregnancy. Commonly used lipid-lowering medications, such as statins or fibrates, have been restricted in pregnant patients due to their potential teratogenic effects [27]. When serum TG levels in pregnancy exceed cut-off points, pregnant women should receive dietary interventions and lifestyle management from obstetricians and nutritionists. Regular measurements and evaluations of blood lipids are essential to improve gestational and long-term health.

For the first time, the study observed the association between gestational TG and postpartum hypertriglyceridemia in women stratified by pre-BMI and GDM. Interestingly, TG values recorded during the first and third trimesters place normal pre-BMI women at higher risk for postpartum hypertriglyceridemia. Over the years, we have been discussing the dangers of overweight and obesity [28], but the present results suggested that the risk of increased gestational TG on postpartum health was even greater for normoponderal women. Similarly, an increased risk of postpartum hypertriglyceridemia was found in women without GDM but not in women with GDM. The increase in TG concentration potentially leads to elevation of FFAs, which might decrease insulin sensitivity and consequently cause a vicious circle between elevated TG and insulin resistance [29, 30]. Women with GDM have lipid metabolism disorders, which may weaken some risks of adverse outcomes of elevated TG. In addition, treatments and nutritional interventions for pregnant women after the diagnosis of GDM may be responsible for the reduced risk of postpartum hypertriglyceridemia. The study suggests that pregnant women with normal pre-BMI and normal pregnancies should pay more attention to the monitoring of serum lipids throughout gestation.

### Study strength and limitations

This is a population-based prospective cohort study concerning the association between serum TG levels during pregnancy and the risk of postpartum hypertriglyceridemia. The description of the longitudinal change in maternal TG both during pregnancy and after delivery overcomes limitations of existing studies that only studied pregnancy. This study was the first to find the association between gestational TG and its elevation and postpartum hypertriglyceridemia, considering the effects of time trajectory and repeated measurements and scientifically reflecting the association. TG cut-off points for the first, second, and third trimesters were found by using modern statistical methods, and they met clinical significance and provided referable guidance on blood lipid control for clinical obstetricians and pregnant women. Another highlight of the study is the comparisons of postpartum TG outcomes among pregnant women with distinct pre-BMI and between women with and without GDM. The results should make pregnant women aware of the importance of gestational lipid control for the sake of their postpartum lipid health.

However, some limitations of the study should be pointed out. First, the follow-up visit was up to 42 days postpartum, and there was no long-term follow-up for the study group. Second, random serum samples were collected at 42 days postpartum rather than fasting samples, which may have led to approximately 0.2–0.3 mmol/L higher serum TG levels, according to several large population studies [11, 31, 32]. Thus, postpartum hypertriglyceridemia was defined as P75 instead of a certain TG level to make the results more reliable. Moreover, the lifestyles of pregnant women, including dietary structures and physical activities that could be important factors for serum TG, need to be considered during pregnancy and after delivery.

## Conclusions

In conclusion, serum TG levels increased with gestational age and decreased at 42 days postpartum. Gestational TG and its elevation during pregnancy were both important risk factors for postpartum hypertriglyceridemia in pregnant women. Serum TG levels should be controlled below 1.93 mmol/L, 2.35 mmol/L, and 3.08 mmol/L in the first, second, and third trimesters to reduce the risk of postpartum hypertriglyceridemia. Moreover, the risk of gestational TG on postpartum hypertriglyceridemia was more significant among pregnant women with normal pre-BMI and without GDM. The study highlights the importance of measurements and evaluations of maternal TG during pregnancy and provides a reference value for clinical serum lipid measurements and follow-up treatments, which can be used for gestational lipid management and prevention of postpartum hypertriglyceridemia.

## Supplementary information

Additional file 1 Title of data: **Table S1** Correlations between TG at 42 days postpartum and gestational TG. Description of data: **Table S1** shows the results of Pearson correlation analysis at the “Associations between serum TG levels during pregnancy and at 42 days postpartum” part of RESULTS. The supplemental file can be provided online to give readers additional information about the work.

Additional file 2 Title of data: Certificate of language editing from American Journal Experts.

## Data Availability

The datasets analysed during the current study are not publicly available due the data also forms part of an ongoing study, but are available from the corresponding author on reasonable request.

## Abbreviations

TG: triglyceride
GDM: gestational diabetes mellitus
PE: preeclampsia
ICP: intrahepatic cholestasis of pregnancy
LGA: large for gestational age
CVD: cardiovascular disease
BMI: body mass index
pre-BMI: prepregnancy body mass index
GWG: gestational weight gain
IADPSG: the International Association of the Diabetes and Pregnancy Study Groups
OGTT: oral glucose tolerance test
TC: total cholesterol
LDL-C: low density lipoprotein cholesterol
HDL-C: high density lipoprotein cholesterol
LME: linear mixed-effect model
BLUP: best linear unbiased predictor
OR: odds ratio
CI: confidence interval
ROC curve: receiver operating characteristic curve
AUC: area under the ROC curve
FFA: free fatty acids
