# Supplementary material for: Changes in serum TG levels during pregnancy and their association with postpartum hypertriglyceridemia: a population-based prospective cohort study
Source: Lipids Health Dis. 2021 Sep 29;20:119. doi: 10.1186/s12944-021-01549-y (PMC8480071; doi:10.1186/s12944-021-01549-y)
Supplement: Supplementary file 1 — Title of data: Table S1 Correlations between TG at 42 days postpartum and gestational TG. Description of data: Table S1 shows the results of Pearson correlation analysis at the “Associations between serum TG levels during pregnancy and at 42 days postpartum” part of RESULTS. The supplemental file can be provided online to give readers additional information about the work. [file 12944_2021_1549_MOESM1_ESM.docx]

**Supplementary Appendix**

This appendix has been provided by the authors to give readers additional information about their work.

Supplement to: Yandi Zhu, et al. Changes in serum TG levels during pregnancy and their association with postpartum hypertriglyceridemia: a population-based prospective cohort study.

| **Table S1** Correlations between TG level at 42 days postpartum and gestational TG level | | | | |
| --- | --- | --- | --- | --- |
|  | Week 6–8 | Week 16 | Week 24 | Week 36 |
| *r* | 0.456 | 0.473 | 0.463 | 0.350 |
| *P* | **<0.001** | **<0.001** | **<0.001** | **<0.001** |
| Dependent variable: serum TG level at 42 days postpartum  Independent variables: serum TG level at each gestational week  *TG* triglyceride | | | | |
