# Supplementary material for: Changes in serum TG levels during pregnancy and their association with postpartum hypertriglyceridemia: a population-based prospective cohort study
Source: Lipids Health Dis. 2021 Sep 29;20:119. doi: 10.1186/s12944-021-01549-y (PMC8480071; doi:10.1186/s12944-021-01549-y)
Supplement: Supplementary file 2 — Title of data: Certificate of language editing from American Journal Experts. [file 12944_2021_1549_MOESM2_ESM.pdf]

AJE®

Editing Certificate

This document certifies that the manuscript

Changes in serum TG levels during pregnancy and their association with postpartum hypertriglyceridemia: a population-based prospective cohort study

prepared by the authors

Yandi Zhu, Haiyan Zhu*, Qinyu Dang, Qian Yang, Dongxu Huang, Yadi Zhang, Xiaxia Cai, Huanling Yu*

was edited for proper English language, grammar, punctuation, spelling, and overall style by one or more of the highly qualified native English speaking editors at AJE.

This certificate was issued on **September 6, 2021** and may be verified on the AJE website using the verification code **6F4E-DD6A-3F32-AF46-92BC**.

Neither the research content nor the authors' intentions were altered in any way during the editing process. Documents receiving this certification should be English-ready for publication; however, the author has the ability to accept or reject our suggestions and changes. To verify the final AJE edited version, please visit our verification page at aje.com/certificate. If you have any questions or concerns about this edited document, please contact AJE at support@aje.com.

AJE provides a range of editing, translation, and manuscript services for researchers and publishers around the world. For more information about our company, services, and partner discounts, please visit aje.com.
